# Large meta-analysis of multiple cancers reveals a common, compact and highly prognostic hypoxia metagene

**DOI:** 10.1038/sj.bjc.6605450

**Published:** 2010-01-19

**Authors:** F M Buffa, A L Harris, C M West, C J Miller

**Affiliations:** 1 Weatherall Institute of Molecular Medicine, University of Oxford, Oxford, OX3 9DS, UK; 2 School of Cancer and Imaging Sciences, The University of Manchester, Manchester, M13 9PT, UK; 3 Paterson Institute for Cancer Research, The University of Manchester, Manchester, M20 4BX, UK

**Keywords:** hypoxia, gene expression, meta-analysis, distant relapse

## Abstract

**Background:**

There is a need to develop robust and clinically applicable gene expression signatures. Hypoxia is a key factor promoting solid tumour progression and resistance to therapy; a hypoxia signature has the potential to be not only prognostic but also to predict benefit from particular interventions.

**Methods:**

An approach for deriving signatures that combine knowledge of gene function and analysis of *in vivo* co-expression patterns was used to define a common hypoxia signature from three head and neck and five breast cancer studies. Previously validated hypoxia-regulated genes (seeds) were used to generate hypoxia co-expression cancer networks.

**Results:**

A common hypoxia signature, or metagene, was derived by selecting genes that were consistently co-expressed with the hypoxia seeds in multiple cancers. This was highly enriched for hypoxia-regulated pathways, and prognostic in multivariate analyses. Genes with the highest connectivity were also the most prognostic, and a reduced metagene consisting of a small number of top-ranked genes, including *VEGFA*, *SLC2A1* and *PGAM1*, outperformed both a larger signature and reported signatures in independent data sets of head and neck, breast and lung cancers.

**Conclusion:**

Combined knowledge of multiple genes' function from *in vitro* experiments together with meta-analysis of multiple cancers can deliver compact and robust signatures suitable for clinical application.

Gene-expression studies attempt to extrapolate biologically and clinically relevant hypotheses from gene expression patterns. However, many current studies make little use of existing knowledge such as gene function within specific pathways, and prognostic signatures are often derived with no reference to the functional roles of their components.

One increasingly popular method that aims to make use of prior knowledge is gene set enrichment analysis (GSEA) (Subramanian *et al*, 2005). It first conducts a supervised analysis by ranking genes according to their ability to discriminate between different sample groups, and then maps them onto previously defined gene sets, typically formed according to common function using annotation sources. The goal is to identify sets containing a statistically significant number of highly ranked genes, and then to use this information to provide functional characterisations for the samples in question. Although powerful, GSEA relies on stratification of the experimental samples into distinct groups, often making it unsuitable for use with heterogeneous clinical data sets.

Another approach often applied to microarray data involves creation of a co-expression network within which each ‘node’ represents a gene, and ‘edges’ are created between genes when their expression patterns are significantly correlated. Co-expression networks have been used to formulate functional and clinical hypotheses from *in vivo* data (Butte and Kohane, 2003; Hahn and Kern, 2005; Wolfe *et al*, 2005). A disadvantage with the approach is that it can be susceptible to the multiple testing issues that arise due to the large number of genes represented on a typical microarray. Setting a low threshold for a significant correlation between genes will result in the inclusion of many spurious links, whereas a high threshold will control the false-positive rate at the expense of omitting many genuine edges.

Here we illustrate and validate a network-based approach with parallels to both GSEA and co-expression networks; for a workflow of the method see Supplementary Material and Methods. It can be applied directly to clinical data, even when the samples cannot be partitioned in advance into distinct groups. The algorithm begins with a collection of ‘seed’ genes that are then used as starting point from which to build an association network. Rather than simply connect gene pairs with high correlation between their expression profiles, the approach defines a ‘neighbourhood of co-expression’ around each seed gene, and then connects seeds that have a significant degree of overlap between their neighbourhoods. This approach is relatively robust against the inclusion of spurious edges, as edges are only added when there is consistently high correlation to many intermediate genes that form the intersection between seeds. We previously used a seed-based approach successfully to predict hypoxia-related genes (Winter *et al*, 2007); this study develops the method in a meta-analysis context to produce robust signatures requiring fewer genes, making them more suitable for clinical use, for example in quantitative RT-PCR analyses of biopsies at presentation.

Hypoxia has a key role in defining the behaviour of many cancers including head and neck squamous cell carcinomas (HNSCCs) (Nordsmark *et al*, 2005) and breast carcinomas (BCs) (Fox *et al*, 2007); thus the identification of common hypoxia-regulated genes is important both for understanding of cancer evolution, and for improved prognosis or development of novel therapies. The described approach was applied to a large meta-analysis of HNSCCs and BCs to define successfully a common and robust hypoxia signature.

## Materials and methods

### Seed clustering

The process begins with *k* seed genes, ∏={*π*_1_,*π*_1_,…*π*_*k*_} (‘gene’ is used throughout for convenience, although ‘transcript’ is generally more accurate). Spearman's correlation, *ρ*, is computed between seeds and genes *Y*={*y*_1_, *y*_2_,…*y*_*m*_} in a data set of *n* samples, *X*={*x*_1_, *x*_2_,…*x*_*n*_}. For each seed/gene pair, their ‘affinity’ is defined as: 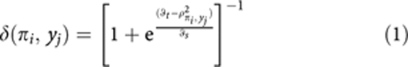 where *θ*_t_ and *θ*_s_ define extent and sharpness of the cluster. When *θ*_s_ → 0, *δ* reduces to the step function with *δ* =0 if *ρ*^2^<*θ*_t_, *δ*=1 if *ρ*^2^>*θ*_t_. In this limit, the method is parameter free, and this will be used in this study. *θ*_t_ is defined objectively using a probability threshold, *α*, of observing a given correlation if the null hypothesis (i.e. no association) was true. This needs to be corrected for multiple testing (Hastie *et al*, 2001) to account for the size of *Y*; here, *α*=0.05 after Bonferroni correction was considered. Finally, a membership function is defined: 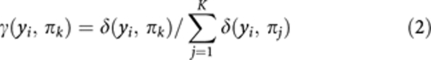


An increasing *γ* indicates stronger membership of a gene to a seed cluster.

### Shared neighbourhood

The shared neighbourhood, *S*, between two seeds is defined as: 
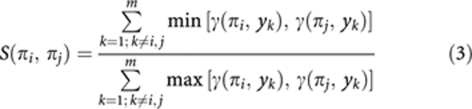
 where *γ* is the membership (Eq. 2). Two seeds are considered to carry a high degree of related information if their clusters share many genes (high *S* values). A sign function is also defined: 
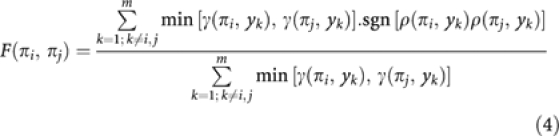
 where sgn(*x*) is the sign function: sgn(*x*)=1 if *x*>0, sgn(*x*)=−1 if *x*<0. If two seeds are correlated with their shared features in the same direction, *F*=1 (seeds are fully concordant); if they are correlated with their shared features in opposite direction, *F*=−1.

### Seed-dependent connectivity

The strength of the relationship between a gene and the whole set of seeds is estimated using the connectivity function: 
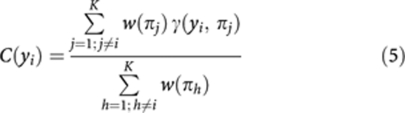
 where *γ* is defined in Eq. 2 and *w* are weights that regulate the importance of each seed. In this study, we consider *w*=1, unless *y*_i_ is one of the seeds, or a probe set biding to the same transcript as the seed; in this case, to avoid bias, *w*=0 for that seed.

A connectivity score is defined as the fractional rank of *C*; that is the ranking normalised between 0 (lowest *C*) and 1 (highest *C*).

### Bootstrapping, Monte Carlo and meta-connectivity score

Random sets of seeds are generated by Monte Carlo sampling, clusters are aggregated around them, and *C* and *S* are calculated. This procedure is repeated to generate null distributions and it provides an estimate of the probability of observing by chance a given value of *C* and *S*.

Bootstrapping is re-sampling with replacement of the original population; it is used to provide maximum likelihood best estimates when an analytical approach is not feasible (Hastie *et al*, 2001). Here, it is used to provide best estimates and confidence limits for *C* and *S*. These are used in a meta-analysis across several data sets to define a meta-connectivity score as: 
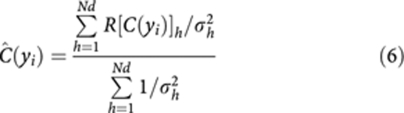
 where R[*C(y*_*i*_)]_*k*_ is the fractional rank of *C* (Eq. 5), *N*_d_ is the number of datasets, *σ*^2^_*k*_ is the variance of the ranked *C*, R[*C(y*_*i*_)]_*k*_, in dataset *k* for gene *y*_i_.

A common metagene between tumour types is derived by taking the *Ĉ* scores product, *πĈ*. This is effectively a rank product, as *Ĉ* is an average rank (Eq. 6).

### Cumulative forest plots based on connectivity score

A summary expression score, *E*, is defined in each sample as the median of the absolute expression of the genes in the signature. The median is used as summary statistics to reduce the effect of outliers. A cumulative forest plot is defined: genes are added to the signature, one by one, in order of their connectivity, *C*, score so that genes that are introduced first have the highest connectivity. At each step, a summary expression, *E*, is derived using the new gene and genes from the previous steps. Samples are then ranked by their *E* value; this assigns a hypoxia score (HS) from lowest (least hypoxic) to highest (most hypoxic). Hypoxia score is then re-normalised between 0 and 1; introduced into a Cox multivariate analysis that includes the other significant clinical covariates and the hazard ratio (HR) of the HS is calculated.

### Data sets, data processing and annotation

NCBI Gene Expression Omnibus (http://www.ncbi.nlm.nih.gov/geo/) was searched for gene expression studies in cancer, published in peer-reviewed journals, where microarray were performed on frozen material extracted before chemotherapy, radiotherapy or adjuvant treatment. Eight data sets (Table 1) were selected that used similar platforms (Affymetrix U133A, B and plus2, www.affymetrix.com). Processing was performed using ‘simpleaffy’ (Wilson and Miller, 2005); the ‘gcrma’ function was used to estimate expression values, data were quantile-normalised and logged (base2). Other data sets were identified for validation in which different technologies were used (Table 1); non-Affymetrix data sets were processed as described in the original publications. More details on pre-processing and annotation are given in the Supplementary Methods.

## Results

### Derivation of a hypoxia expression network

**Figure 1:**
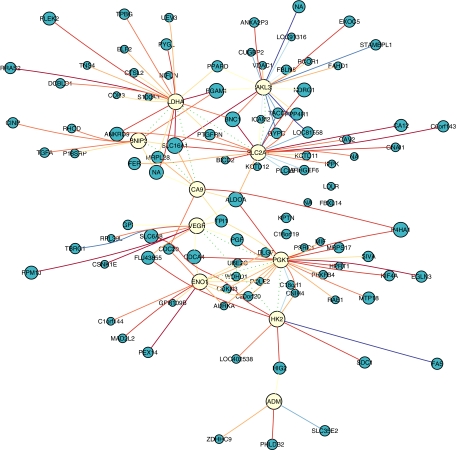
Hypoxia gene-expression network in HNSCC (Vice 125 data set). Seeds (yellow) and learnt genes (blue) are shown; circle size is proportional to *C* score. Genes with top 20% *C* scores are shown. Solid edges connect cluster members with seeds; length is proportional to membership, colour represents Spearman correlation (blue, −1; red, +1). Green dotted edges connect seeds; their length is proportional to the shared neighbourhood, *S*. This figure appears in colour in the HTML version.

**Figure 2:**
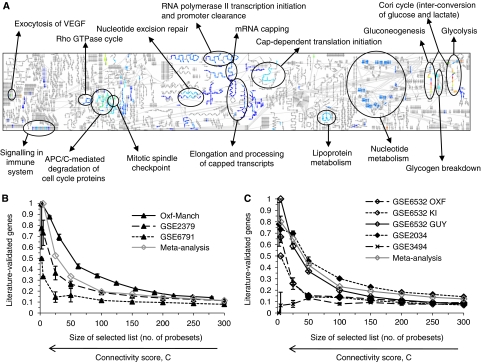
Hypoxia network mapped onto Reactome pathways (**A**) coloured by increasing *C* score from dark blue to bright red; and validation of up-regulated HNSCC (**B**) and BC (**C**) signatures by comparison with the literature. The proportion of literature-validated genes is shown as function of the number of top-ranked (by *C* score) genes considered; standard errors estimated by bootstrap. This figure appears in colour in the HTML version.

A hypoxia expression network was built first in a data set comprising 59 HNSCC tumour samples (Vice 125; Table 1) using well-characterised hypoxia-related genes identified from the literature covering a comprehensive set of hypoxia-induced pathways (set A, Supplementary Table S1). These were adrenomedullin (*ADM*), adenylate kinase 3-like 1 (*AK3L1*), BCL2/adenovirus E1B 19 kDa interacting protein 3 (*BNIP3*), carbonic anhydrase IX (*CA9*), enolase 1 (*ENO1*), hexokinase 2 (*HK2*), lactate dehydrogenase A (*LDHA*), phosphoglycerate kinase 1 (*PGK1*), solute carrier family 2 member 1 (*SLC2A1*) and solute carrier family 2 (*VEGFA*). The resultant network (Figure 1) was observed to map distinct regions of the Reactome (www.reactome.org) network and several hypoxia-related pathways (Figures 2; Supplementary Figure S1). The method was applied to additional HNSCC and BC training data sets (Table 1) with similar results (Supplementary Table S2).

In the resulting expression networks, high shared neighbourhood, *S* (Eq. 3), values between seed pairs were generally associated with a high pair-wise correlation. However, this relationship did not always hold. An example is given in Supplementary Figure S2, where genes in a published 245-gene literature list (LL) (Winter *et al*, 2007) were used as starting seeds. Many of the seeds with high pair-wise *S* but low correlation appeared in the same KEGG (http://www.genome.jp/kegg/) pathway but could not be detected in a straightforward correlation analysis (Supplementary Figure S2). Furthermore some seeds showed markedly different *in vivo* and *in vitro* behaviours; for example, *PFKFB3* (set B, Supplementary Table S1) did not have significant overlap with any other seeds, whereas *CCNG2* showed a consistent inverse correlation with other seeds (*F*<0; Eq. 4), supporting results from previous studies (Choi and Chen, 2005). Thus, the method was able to identify seeds that behave differently from their peers; for the rest of this study, only the conservative seed set A was used. This set showed higher pair-wise *S* values than any other set of randomly selected seeds (repeated 1000 times) from the 245-gene LL.

### Seed-dependent connectivity identifies a hypoxia signature

Genes in the co-expression networks were ranked by their connectivity score, *C* (Eq. 5), and compared with the hypoxia 245-gene LL. As the latter is biased towards up-regulated genes (Harris, 2002), only genes showing consistent positive correlation with the initial seeds were considered. To avoid bias, the initial seeds were excluded from this comparison. The relative proportion of known hypoxia genes increased with increasing connectivity, *C*, score (Figure 2), confirming its benefit as a metric for predicting functional relationships. Similar results were observed with different clustering and pre-processing methods (Supplementary Figure S3). However, differences were observed between data sets. Much of this inter-experimental variation is likely to reflect differences in both the patient populations and the processing of the biological material. For example, both data sets GSE6791 and GSE3494, which showed a lower level of enrichment for hypoxia genes than others, featured samples with the highest proportions of tumour cells selected either by microdissection or visual scoring.

Next we selected a subset of ‘hub’ genes from the hypoxia network, with the goal of using them as a hypoxia signature. Genes with high connectivity, *C* (Eq. 5), score (*P*<0.01, estimated by Monte Carlo simulation) were considered (Supplementary Table S2). Each of these genes had a greater-than-expected overlap with the neighbourhoods of all other genes in the hypoxia network (Supplementary Figure S4). The seeds were only selected if they were hubs with respect to all other seeds. Using the Reactome database, we confirmed that pathways known to be regulated by hypoxia, such as glycolysis, gluconeogenesis, glucose metabolism and Cori cycle (recycling of lactic acid), were consistently over-represented in these genes (Figure 2; Supplementary Table S3). Similarly, GO analysis (http://genecodis.dacya.ucm.es) found over-representation (false discovery rate <0.05) of pathways such as glycolysis, phosphoinositide-mediated signalling, nuclear mRNA splicing, translational initiation, regulation of cell cycle, ubiquitin-dependent protein catabolism, apoptosis and regulation of cell proliferation. Over-represented molecular functions included ATP binding, nucleotide binding, lipoic acid binding, oxidoreductase and L-lactate dehydrogenase activity.

### Meta-signature enrichment and the prognostic value of compact signatures

We selected genes that showed consistent high connectivity across data sets and derived meta-signatures for hypoxia in HNSCC and BC. Interestingly, although some of the data sets performed poorly on their own, meta-analysis signatures were robust to their inclusion and performed well (Figures 2B and C).

We assessed the prognostic relevance of meta-signatures in four independent data sets (Table 1). Samples were ranked using a summary expression score, *E*, of the genes in the signature; this produced a hypoxia score, which assigns a hypoxic status to the tumours in the validation data sets. Multivariate Cox analysis including available clinical factors was carried out using each data set; clinical variables were selected using backward-stepwise maximum likelihood. The HS was introduced into the reduced clinical model to estimate the prognostic significance of the meta-signatures independently from other clinical variables (Supplementary Figure S5 and Table S4).

To address whether smaller signatures with equal prognostic ability could be derived by using a more stringent *C* score, cumulative forest plots were generated in which genes were introduced into the HS calculation one by one, in decreasing order of their meta-*Ĉ* score (Supplementary Figure S5). Only a few genes were needed before the HR stabilised and a reduced signature was found to be at least as prognostic as a larger one (Supplementary Figure S5 and Table S4). Interestingly, when genes were introduced into the cumulative plots in random order, rather than by their ranked *Ĉ* score, more genes were needed to reach equivalent prognostic significance (Supplementary Figure S5).

### A common hypoxia metagene across cancer types

Common hubs in HNSCC and BC were selected by considering, for each gene, the product, *πĈ*, of the *Ĉ* scores between the HNSCC and BC meta-analyses. A common metagene was derived by considering genes with *πĈ*>0.5 (Table 2; Supplementary Table S5). This hard cut-off was chosen because a gene with a *πĈ* score approaching that which would be expected by chance (*πĈ*≈0.5) in one tumour site would have to achieve a maximal score in the other tumour site to be included.

**Figure 3:**
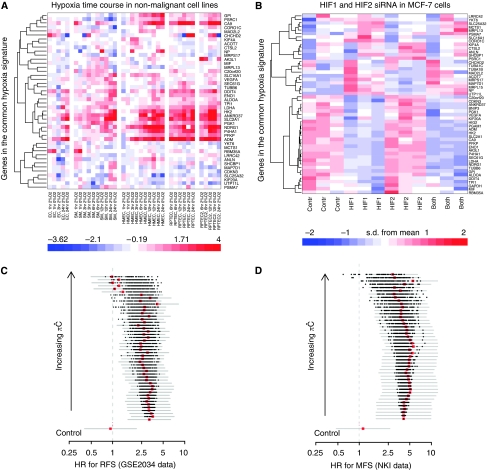
Common hypoxia signature of 51 genes. (**A**) Hypoxia/normoxia expression ratio in endothelial, smooth muscle, human mammalian epithelial, renal proximal tubule epithelial cells (EC, SMC, HMEC, RPTEC); and in (**B**) HIF1a/HIF2a siRNA experiment. (**C**, **D**) Connectivity-ranked forest plots: metastases- and recurrence-free survival (MFS, RFS) hazard ratio (HR) (red) with 95% confidence intervals, and HRs if permuted list (black). Control: random sampling of *N*=51 genes ( × 100 resampling).

We investigated in cell lines potential regulation of genes in the common metagene by hypoxia and by HIF1a, the main mediator of the hypoxia response in cancer. We considered two data sets: a hypoxia time course in a panel of epithelial and endothelial non-malignant cells (Chi *et al*, 2006), and an HIF1a and HIF2a siRNA experiment in MCF7 BC cells (Elvidge *et al*, 2006) exposed to hypoxia. For details of these data we refer to the original publications. Although differences between cell lines and BC *in vivo* are expected, a high proportion of genes in the common metagene (38 out of 51) showed either regulation in the hypoxia time course or in the siRNA experiment (Figure 3A, B; Supplementary Table S5). Several of these genes were also predicted as HIF1a targets and showed potential HIF1a binding sites (Supplementary Table S5). Furthermore, 22 had already been found hypoxia regulated by previously published report (Supplementary Table S5). Overall approximately 80% (42 out of 51) of genes in the common metagene were confirmed by at least one validation, several of them by more than one.

The common hypoxia metagene was prognostic in independent data sets of different cancer types (Table 3) and showed greater prognostic power than (1) an *in vitro* derived hypoxia signature (Chi *et al*, 2006), (2) the initial seeds and (3) our 99-gene HNSCC hypoxia metagene derived previously (Winter *et al*, 2007). A signature derived by selecting genes co-expressed with VEGF in BC (Desmedt *et al*, 2008) had no independent prognostic significance (data not shown), in agreement with the published study. In a further validation using Oncomine (http://www.oncomine.org), all but one of the 15 top-ranked (by *π*Ĉ score) genes showed prognostic significance in at least one tumour site (*P*<0.0001). The only top gene for which prognostic significance was not reported in Oncomine, SLC2A1 (*GLUT1*), is prognostic in other studies (Oliver *et al*, 2004).

Finally, cumulative forest plots based on connectivity score (Figure 3) showed no further improvement in HR after addition of a small number of genes. Although differences were observed between HNSCC, BC and lung cancers, we found in all cases that a common signature reduced to a small number of *π*Ĉ score top-ranked genes was at least as prognostic as the full signature (Figure 3C, D; Table 3).

## Discussion

Hypoxia is a frequent feature of poor-prognosis tumours, and the identification of common *in vivo* hypoxia-related genes is desirable both for prognostic stratification of patients and development of novel therapies. Although prognostic markers of hypoxia have been identified, there are discrepancies between studies and powerful methods used in large meta-analyses are needed to define generally applicable signatures. A method is described for defining a hypoxia signature that combines previous knowledge derived from *in vitro* experiments, with co-expression data produced from *in vivo* samples. We show that by constructing a gene expression network and then extracting core ‘hub’ (high connectivity) genes it is possible to define signatures that are significantly enriched for phenotype-specific genes, and pathways. Although we have used this method to derive a compact and clinically relevant signature of hypoxia in cancer, the approach is likely to have broader applicability.

Specifically, we used the described method in a meta-analysis of 1136 HNSCCs and BCs to derive tissue-specific and common signatures of hypoxia by including only genes that are consistently useful across multiple experiments or tissue types. The ability of the method to derive highly prognostic hypoxia signatures despite differences between data sets highlights its robustness.

The gene expression network used to construct the signature was found to be biologically relevant and to map to a discrete set of biochemical pathways, which is significantly enriched for hypoxia-regulated genes and pathways. This finding highlights that not only *in vitro* data can assist understanding of clinical data, but also the reverse, that clinical data can be used to formulate specific biological hypotheses.

Remarkably, a reduced common hypoxia metagene containing as few as three genes, namely *VEGFA*, *SLC2A1* and *PGAM1*, was as prognostic as a large signature in independent BC and HNSCC series. Furthermore, it was more prognostic than several reported signatures when tested in a set of independent data sets, suggesting a level of general applicability. Specifically, genes with highest connectivity were also the most prognostic across a panel of cancers. This further validates the method, as prognosis was not used to select genes that were only ranked by their connectivity; and this ranking was derived in independent data sets. Although a reduced signature was prognostic in all tumour sites tested, the number of genes before convergence was lower in HNSCC and BC than lung cancer. This offers another positive control as this was a common signature between HNSCC and BC, thus it is expected to reflect their biology to a better extent; however, it also indicates a degree of tumour specificity. The common signature and the tumour-type-specific signatures are being evaluated in prospective prognostic and predictive studies in HNSCC and breast cancer.

In summary, this study uses information from *in vitro* experiments regarding the function of multiple genes combined with *in vivo* co-expression patterns to derive a common hypoxia metagene in multiple cancers that is highly prognostic, while being compact and robust.

## Figures and Tables

**Table 1:** Data sets used to train and validate the hypoxia signature

**Name**	**Size**	**Site**	**Reference**
*Training data sets*			
Vice125	59	HN	Winter *et al* (2007)
GSE2379	20	HN	Cromer *et al* (2004)
GSE6791	42	HN	Pyeon *et al* (2007)
GSE6532Oxf	149	Breast	Loi *et al* (2008)
GSE6532KI	178	Breast	Loi *et al* (2008)
GSE6532GUY	87	Breast	Loi *et al* (2008)
GSE2034	286	Breast	Carroll *et al* (2006)
GSE3494	315	Breast	Miller *et al* (2005)
			
*Validation data sets*			
NKI	295	Breast	van de Vijver *et al* (2002)
Beer	86	Lung	Beer *et al* (2002)
GSE4573	130	Lung	Raponi *et al* (2006)
Chung	60	HN	Chung *et al* (2004)

Abbreviation: HN=head and neck.

**Table 2:** Top-ranked genes of the common hypoxia metagene

**HGNC symbol**	**Names**	**Pathway (source)**	**Breast ranked score**	**HNSCC ranked score**	**Common score (∏Ĉ)**
*VEGFA*	Vascular endothelial growth factor A	VEGF signalling (KEGG)	0.99	0.99	0.98
*SLC2A1*	Solute carrier family 2, member 1	Adipocytokine signalling (KEGG)	0.99	0.98	0.97
*PGAM1*	Phosphoglycerate mutase 1	Glycolysis/Gluconeogenesis (KEGG)	0.96	1.00	0.96
*ENO1*	Enolase 1	Glycolysis/Gluconeogenesis (KEGG)	0.97	0.98	0.95
*LDHA*	Lactate dehydrogenase A	Glycolysis/Gluconeogenesis (KEGG)	0.94	1.00	0.93
*TPI1*	Triosephosphate isomerase 1	Glycolysis/Gluconeogenesis (KEGG)	0.92	0.99	0.91
*P4HA1*	Prolyl 4-hydroxylase, *α*-polypeptide I	Arginine and proline metabolism (KEGG)	0.83	1.00	0.83
*MRPS17*	Mitochondrial ribosomal protein S17	Transport (GO:0006810)	0.84	0.97	0.82
*CDKN3*	Cyclin-dependent kinase inhibitor 3	G_1_/S transition of mitotic cell cycle (GO:0000082)	0.85	0.95	0.81
*ADM*	Adrenomedullin	Signal transduction (GO:0007165)	0.74	1.00	0.74
*NDRG1*	N-myc downstream regulated 1	Response to metal ion (GO:0010038)	0.71	0.99	0.71
*TUBB6*	Tubulin, *β*6	Gap junction (KEGG)	0.85	0.84	0.71
*ALDOA*	Aldolase A, fructose-bisphosphate	Glycolysis/Gluconeogenesis (KEGG)	0.86	0.80	0.69
*MIF*	Macrophage migration inhibitory factor	Tyrosine metabolism (KEGG)	0.71	0.93	0.66
*ACOT7*	Acyl-CoA thioesterase 7	Lipid metabolism (KEGG)	0.73	0.89	0.65

**Table 3:** Prognostic significance of the common hypoxia metagene versus other hypoxia signatures

**Data (Table 1)**	**End point and significant clinical covariates (Cov.)^a^**	***In vitro* hypoxia signature (Chi *et al*, 2006)**	**HN hypoxia metagene (Winter *et al*, 2007)**	**Initial seeds^b^**	**PCA score^c^**	**CHM 51genes**	**Reduced CHM^d^ k genes**
NKI	*End point:* MFS * Cov*.: Age, tumour size, nodal status, grade, adj. treatment	2.94 (1.39, 6.23) *P*=0.005	3.58 (1.53, 8.39) *P*=0.003	2.41 (1.05, 5.53) *P*=0.038	3.22 (1.37, 7.56) *P*=0.007	4.15 (1.73, 9.96) *P*=0.002	5.58 (2.41, 12.90) *P*<*0.001*, *k*=3
							
GSE2034^e^	*End point:* RFS * Cov.:* NA	2.20 (1.11, 4.34) *P*=0.024	1.92 (0.97, 3.78) *P*=0.061	2.36 (0.95, 3.77) *P*=0.014	1.98 (1.01, 3.90) *P*=0.048	3.22 (1.63, 6.35) *P*=0.001	4.15 (2.10, 8.18) *P*<*0.001*, *k*=10
							
GSE3494^e^	*End point:* DSS * Cov.:* ER, PgR, tumour size, nodal status	1.19 (0.45, 3.13) *P*=0.732	2.07 (0.77, 5.53) *P*=0.149	2.87 (1.25, 4.49) *P*=0.029	3.61 (1.33, 9.82) *P*=0.012	3.16 (1.05, 9.53) *P*=0.042	4.27 (1.53, 11.94) *P*=0.006, *k*=2
							
Chung	*End point:* RFS * Cov.:* Intrinsic sign., differentiation, batch (strata)	3.06 (0.53, 17.6) *P*=0.210	14.83 (1.8, 122.4) *P*=0.012	6.71 (0.93, 48.4) *P*=0.059	1.25 (0.14, 11.4) *P*=0.840	6.25 (0.83, 47.2) *P*=0.077	34.66 (4.26, 281.95) *P*=0.001, *k*=2
							
Beer	*End point:* OS * Cov.:* Stage	2.59 (1.59, 4.2) *P*=0.829	6.90 (1.34, 35.6) *P*=0.021	3.98 (0.72, 22.0) *P*=0.114	3.45 (0.59, 20.0) *P*=0.168	12.84 (1.71, 96.5) *P*=0.014	24.57 (2.83, 213.36) *P*=0.004, *k*=23
							
GSE4573	*End point:* OS * Cov.:* Nodal status	3.15 (1.32, 7.54) *P*=0.010	1.49 (0.65, 3.43) *P*=0.350	2.31 (0.93, 5.72) *P*=0.070	1.61 (1.14, 2.3) *P*=0.035	2.75 (1.15, 6.56) *P*=0.023	2.90 (1.27, 6.61) *P*=0.012, *k*=38

Abbreviations: CHM=common hypoxia metagene; DSS=disease-specific survival; ER/PgR=estrogen/progesterone receptor; MFS=metastases-free survival; RFS=recurrence-free survival; OS=overall survival.

a Reduced models of clinical covariates are derived using backward-stepwise likelihood. Signature scores are entered into the reduced model; hazard ratio, 95% confidence limits and significance (model with and without the signature) are shown.

b Summary score, *E*, is calculated for the signature including only the initial seeds.

c Score obtained using principal components analysis (Supplementary Methods).

d At convergence in the cumulative forest plots.

e These two data sets were used to develop the signature but no training on outcome was carried out.
