# Large meta-analysis of multiple cancers reveals a common, compact and highly prognostic hypoxia metagene

**DOI:** 10.1038/sj.bjc.6605893

**Published:** 2010-09-07

**Authors:** F M Buffa, A L Harris, C M West, C J Miller

**Correction to**: *British Journal of Cancer* (2010) **102**, 428–435; doi:10.1038/sj.bjc.6605450

Owing to an error during the web publication of this paper, an element of the Supplementary Material, vital to the understanding of the paper, was omitted. This document, ‘Suppl_online_material.pdf’, has now been uploaded to accompany the paper and appears online (please see ‘Correction to’ link above).

The publishers apologise to Dr Buffa and her co-authors for this unfortunate error.

