# Large meta-analysis of multiple cancers reveals a common, compact and highly prognostic hypoxia metagene

**DOI:** 10.1038/sj.bjc.6605933

**Published:** 2010-09-28

**Authors:** F M Buffa, A L Harris, C M West, C J Miller

**Correction to**: *British Journal of Cancer* (2010) **102**, 428–435; doi:10.1038/sj.bjc.6605450

Upon publication of the above paper in early 2010, the authors noticed that a previous version of Supplementary Table S5 had been uploaded. The incorrect version did not rank the genes in order, which was vital to the understanding of their relative significance. The publishers apologise for this error and have now uploaded the correct Table S5 to accompany the paper online.

