# Supplementary material for: Large meta-analysis of multiple cancers reveals a common, compact and highly prognostic hypoxia metagene
Source: Br J Cancer. 2010 Jan 19;102(2):428–35. doi: 10.1038/sj.bjc.6605450 (PMC2816644; doi:10.1038/sj.bjc.6605450)
Supplement: Supplementary Figure S1 [file 6605450x1.pdf]

A

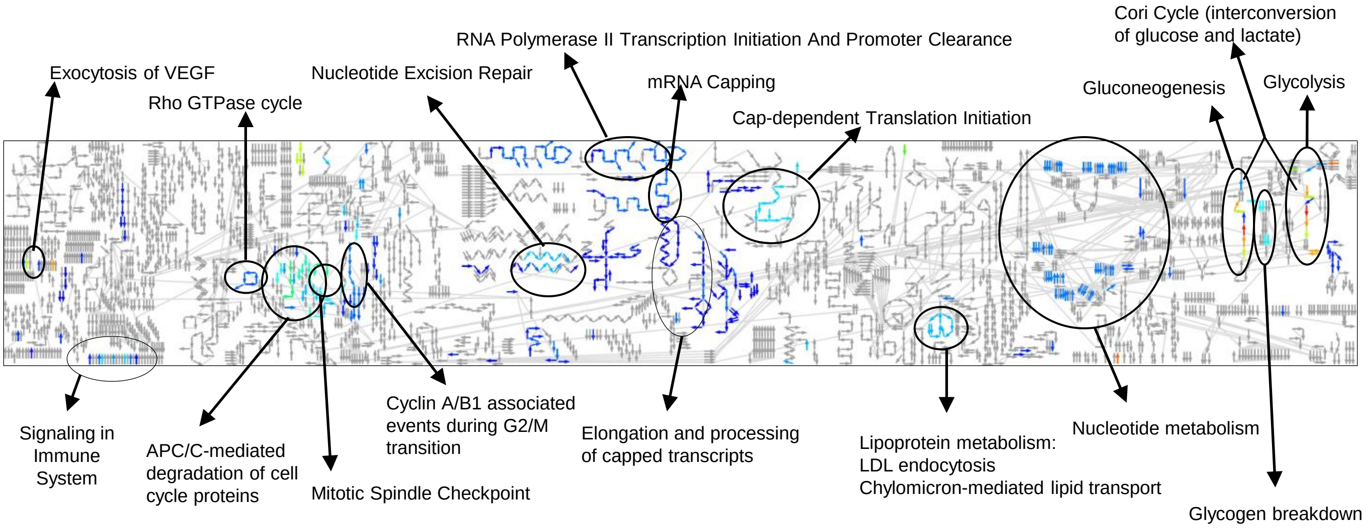

B

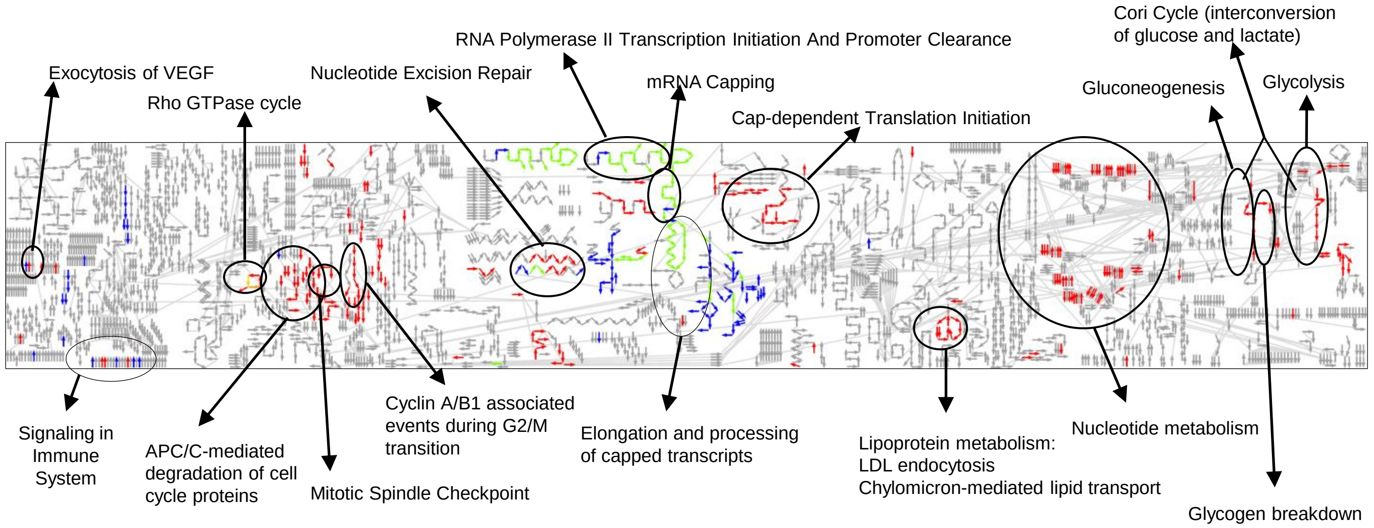

Figure S1. Validation of in-vivo hypoxia signature (HS) using Reactome pathway database. A) The complete chart of the Reactome pathway database (www.reactome.org) is shown with mapping of genes with top-ranked connectivity, C, score in HN Vice125 dataset (Table 1). The names of pathways represented in the signature are shown. Colouring is done according to the average values of all identifiers linked to that reaction. A) Colouring from drak blue to bright red indicates increasing C rank. B) Colouring indicates direction of regulation: consistently up-regulated reactions are in red, consistently down-regulated in blue, green represent reactions where some up-regulated and some down-regulated genes were observed.
