# Supplementary material for: Large meta-analysis of multiple cancers reveals a common, compact and highly prognostic hypoxia metagene
Source: Br J Cancer. 2010 Jan 19;102(2):428–35. doi: 10.1038/sj.bjc.6605450 (PMC2816644; doi:10.1038/sj.bjc.6605450)

Figure S2.The overlap between pairs of seed clusters (ie. the S score) is plotted as a function of the correlation between the expression values for the same pair of seeds. The seeds were set to the ‘literature list’ (http://cancerres.aacrjournals.org/cgi/data/67/7/3441/DC1/1); Vice125 dataset was used (Table 1).

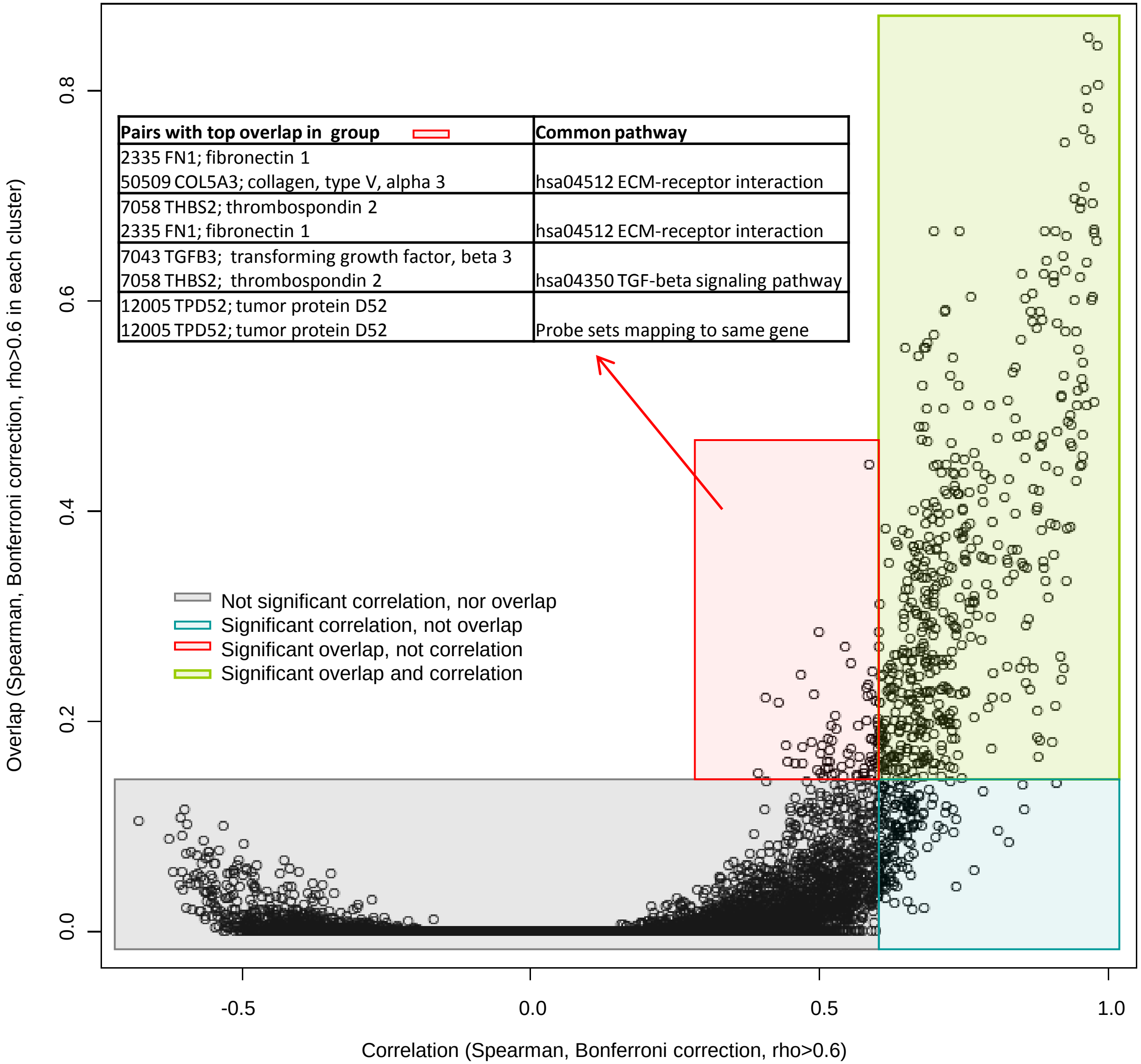

Supplement: Supplementary Figure S2 [file 6605450x2.pdf]
