# Supplementary material for: Large meta-analysis of multiple cancers reveals a common, compact and highly prognostic hypoxia metagene
Source: Br J Cancer. 2010 Jan 19;102(2):428–35. doi: 10.1038/sj.bjc.6605450 (PMC2816644; doi:10.1038/sj.bjc.6605450)
Supplement: Supplementary Figure S3 [file 6605450x3.pdf]

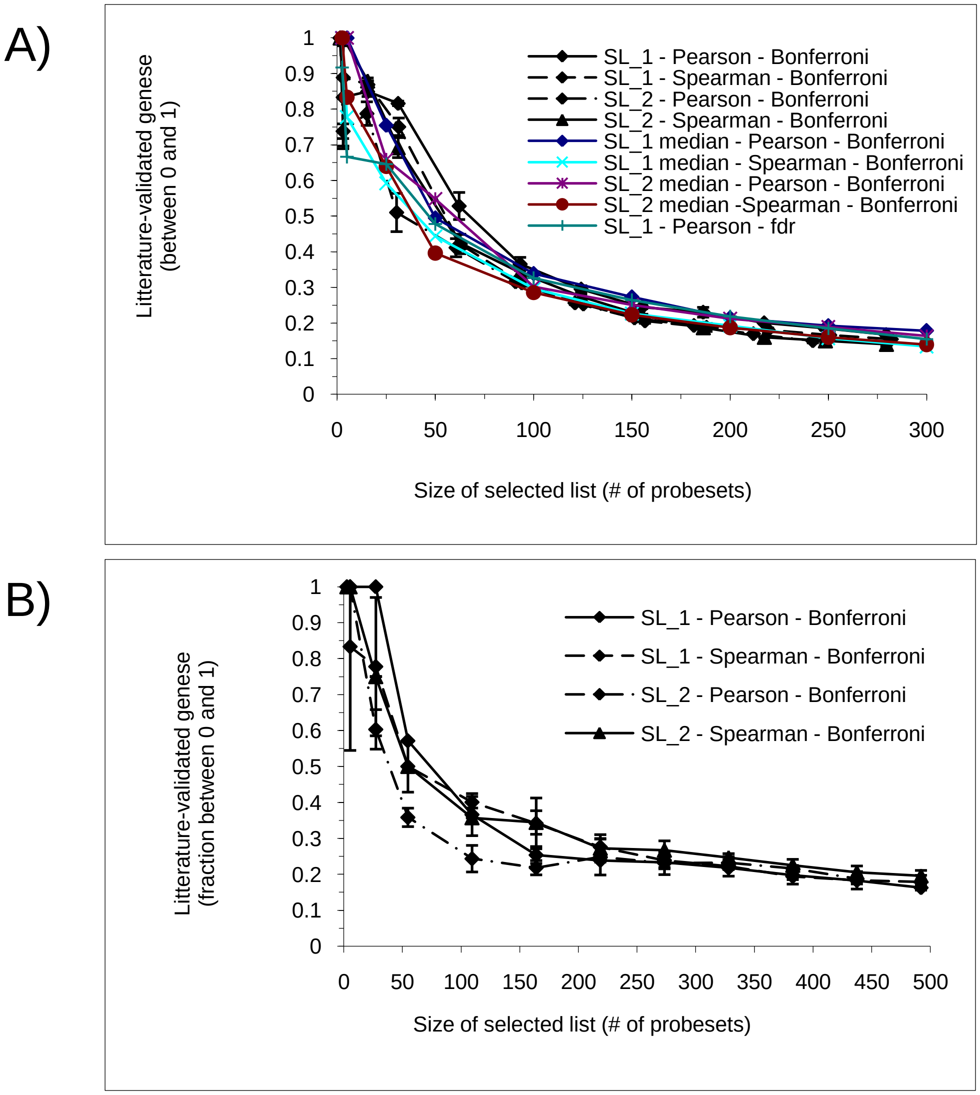

Figure S3. Comparison of the results from the literature validation of the hypoxia signatures obtained using a range of different methods for clustering, multiple test correction, and initial seed choice. The 'literature list' was our literature reference (5). The Vice125 dataset was used (Table 1). Data were pre-processed using GCRMA (A) or MAS5 (B). SL_1 and 2 are respectively set B and A described in Table S1. The attribute "median" indicates that when more than one probeset mapped to the same gene, the "median" criterion was used to assign the expression to the initial seed for that gene rather than the default "best candidate" criterion (see Suppl. Methods section). Pearson or Spearman correlation were used as clustering distance metrics, with either Bonferroni correction for multiple testing or false discovery rate correction permutation of the samples. In all cases data were filtered for unspecific probesets and low expression probesets as indicated in the Suppl. Methods.
