# Supplementary material for: Large meta-analysis of multiple cancers reveals a common, compact and highly prognostic hypoxia metagene
Source: Br J Cancer. 2010 Jan 19;102(2):428–35. doi: 10.1038/sj.bjc.6605450 (PMC2816644; doi:10.1038/sj.bjc.6605450)
Supplement: Supplementary Figure S4 [file 6605450x4.pdf]

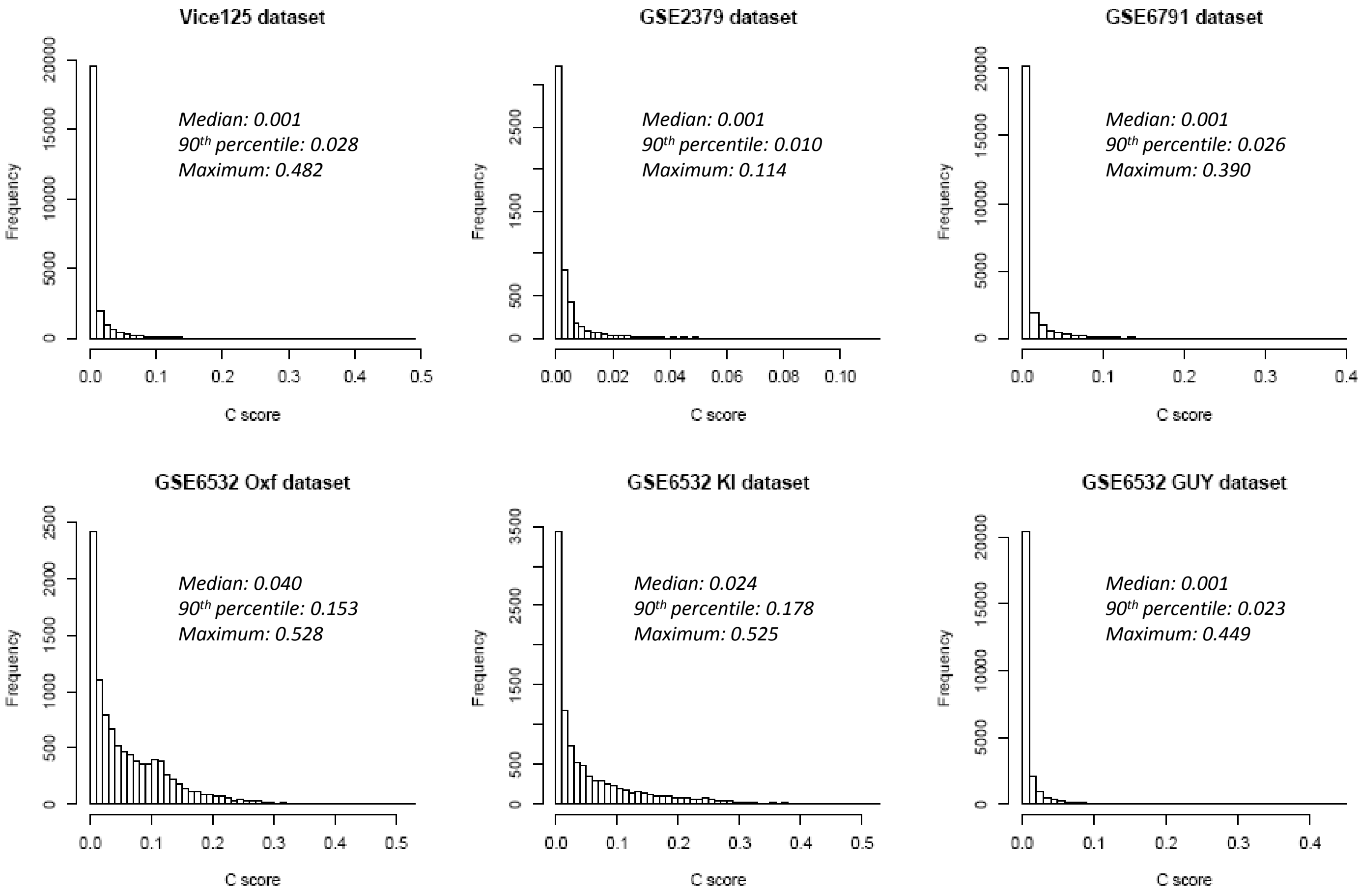

Figure S4. Frequency distributions for the connectivity score C of the hypoxia networks trained in head and neck and breast cancer datasets (Table1). The distribution of the mean values of C after bootstrapping (n=300) is shown for genes on the array that passed initial filtering (see Suppl. Methods). Seed choice A in Table S1.

Comments to Figure S4: properties of connectivity C score

The distribution of C for all genes was found to be highly skewed towards zero in all datasets considered irrespectively of seed choice, filtering, bootstrapping, pre-preprocessing or clustering methods (data not shown). Thus, as expected, most genes represented on the array do not cluster with any of the seeds, and the probability of a gene being a member of one or more of the seed clusters is extremely small. Both skewness and maximum value of the distribution of C varied between datasets; this is due to various factors including the difference in size of the datasets, the difference in population, the difference in size and the size and generation of Affymetrix arrays considered. For example, C was less skewed in GSE6532Oxf and GSE6532KI. These are between two and three times larger than the other datasets (Table 1). It is possible that some true correlations are not found to be significant in the smaller datasets. Furthermore, these two datasets use smaller arrays (Table 1) containing a subgroup of relatively well-characterised transcripts; thus the proportion of transcripts in these arrays which are involved in cancer metabolism-related pathways, and which cluster at least with one of the seeds, might be higher. However, the maximum C score is similar between these and the other datasets suggest that only genes with a lower C score, that is the potential false positives, are missed out, but not the ones with a high C score which are the ones we believe to be the real positive for hypoxia in-vivo. To confirm this, a pair-wise comparison between HG U133a and HG U133-plus2 training datasets (excluding GSE6791 where samples are processed using a different protocol, as discussed in the next sections) of the top-ranked genes showed that the overall overlap between datasets is higher when top C scores were considered (median overlap for genes with $C>0.4$ is 12%) than when lower scores are included (median overlap for genes with $C>0.2$ is 3%).

Different is the case of dataset GSE2379, where a much lower C score maximum is observed. This dataset uses Affymetrix arrays of older generation, and it is much smaller than the other datasets (Table 1), approaching the minimum size needed to apply the present method (when using 20 samples the minimum correlation which can be detected at 0.05 significance level and with a 90% power is $r=0.66$).
