# Supplementary material for: Large meta-analysis of multiple cancers reveals a common, compact and highly prognostic hypoxia metagene
Source: Br J Cancer. 2010 Jan 19;102(2):428–35. doi: 10.1038/sj.bjc.6605450 (PMC2816644; doi:10.1038/sj.bjc.6605450)
Supplement: Supplementary Figure S5 [file 6605450x5.ppt]

## Slide 1
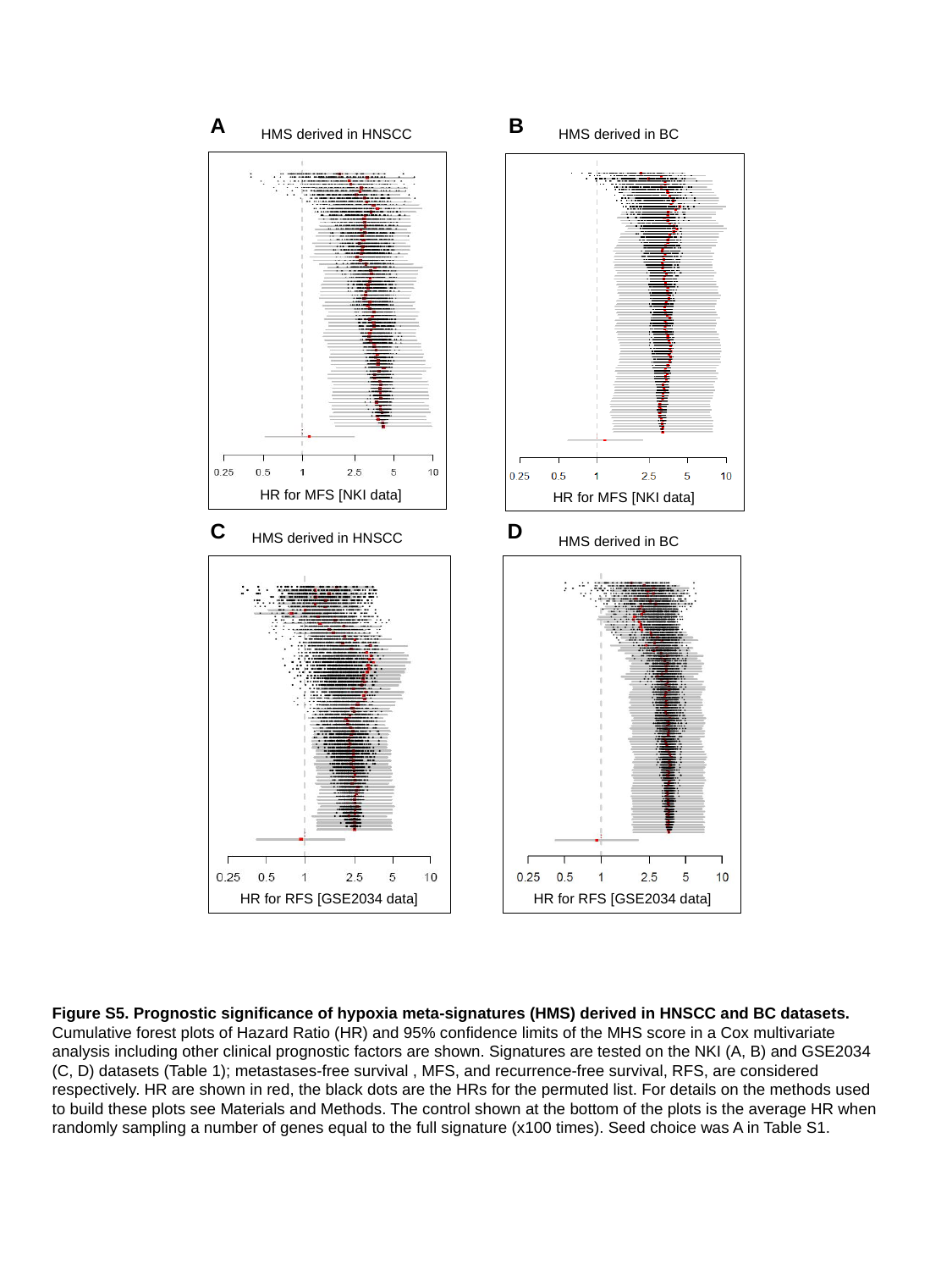

A
B
HMS derived in HNSCC
HMS derived in BC
c)
 HR for MFS [NKI data]
 HR for MFS [NKI data]
C
D
HMS derived in HNSCC
HMS derived in BC
 HR for RFS [GSE2034 data]
 HR for RFS [GSE2034 data]
Figure S5. Prognostic significance of hypoxia meta-signatures (HMS) derived in HNSCC and BC datasets. Cumulative forest plots of Hazard Ratio (HR) and 95% confidence limits of the MHS score in a Cox multivariate analysis including other clinical prognostic factors are shown. Signatures are tested on the NKI (A, B) and GSE2034 (C, D) datasets (Table 1); metastases-free survival , MFS, and recurrence-free survival, RFS, are considered respectively. HR are shown in red, the black dots are the HRs for the permuted list. For details on the methods used to build these plots see Materials and Methods. The control shown at the bottom of the plots is the average HR when randomly sampling a number of genes equal to the full signature (x100 times). Seed choice was A in Table S1.
