# Supplementary material for: Large meta-analysis of multiple cancers reveals a common, compact and highly prognostic hypoxia metagene
Source: Br J Cancer. 2010 Jan 19;102(2):428–35. doi: 10.1038/sj.bjc.6605450 (PMC2816644; doi:10.1038/sj.bjc.6605450)
Supplement: Supplementary Table S1 [file 6605450x6.doc]

**Table S1.** The conservative set of seeds used in the analysis

| **Gene Symbol** | **Long Name** | **Ensembl** | **KEGG** | **Seed Set*** |
| --- | --- | --- | --- | --- |
| ADM | adrenomedullin | ENSG00000148926 |  | A, B |
| AK3L1 | adenylate kinase 3-like 1 | ENSG00000162433 | hsa00230 Purine metabolism | A, B |
| BNIP3 | BCL2/adenovirus E1B 19kDa interacting protein 3 | ENSG00000176171 |  | B |
| CA9 | carbonic anhydrase IX | ENSG00000107159 | hsa00910 Nitrogen metabolism | A, B |
| CCNG2 | cyclin G2 | ENSG00000138764 | hsa04115 p53 signaling pathway | A |
| ENO1 | enolase 1, (alpha) | ENSG00000074800 | hsa00010 Glycolysis / Gluconeogenesis | A, B |
| HK2 | hexokinase 2 | ENSG00000159399 | hsa00010 Glycolysis / Gluconeogenesis | A, B |
| LDHA | lactate dehydrogenase A | ENSG00000134333 | hsa00010 Glycolysis / Gluconeogenesis | B |
| PFKFB3 | 6-phosphofructo-2-kinase/fructose-2,6-biphosphatase 3 | ENSG00000170525 | hsa00051 Fructose and mannose metabolism | A |
| PGK1 | phosphoglycerate kinase 1 | ENSG00000102144 | hsa00010 Glycolysis / Gluconeogenesis | A, B |
| SLC2A1 | solute carrier family 2 (facilitated glucose transporter), member 1 | ENSG00000117394 | hsa04920 Adipocytokine signaling pathway | A, B |
| VEGFA | vascular endothelial growth factor A | ENSG00000112715 |  | A, B |

*Set A is the set used for all the analyses presented in the manuscript; set B has been published previously (Winter *et al*, 2007) and it was compared to set A.
