# Supplementary material for: Large meta-analysis of multiple cancers reveals a common, compact and highly prognostic hypoxia metagene
Source: Br J Cancer. 2010 Jan 19;102(2):428–35. doi: 10.1038/sj.bjc.6605450 (PMC2816644; doi:10.1038/sj.bjc.6605450)
Supplement: Supplementary Table S4 [file 6605450x9.doc]

**Table S4.** Reduced signatures in each validation set and results from Cox multivariate analyses including significant clinical covariates in each set.

| **Study ID**  **Tumour Site**  **(Table 1)*** | **HR of**  **HN**  **Reduced^£^**  **Meta-Signature**  **[± CI]**  **P value**  **Top-K Ĉ scores** | **HR of**  **HN**  **top-99 Ĉ scores**  **Meta-Signature**  **[± CI]**  **P value** | **HR of**  **breast**  **Reduced^£^**  **Meta-Signature**  **[± CI]**  **P value**  **Top-K Ĉ scores** | **HR of**  **breast**  **top-99 Ĉ scores**  **Meta-Signature**  **[± CI]**  **P value** |
| --- | --- | --- | --- | --- |
| GSE2034 | 3.51 [1.79, 6.87] | 2.50 [1.28, 4.88] | 3.78 [1.93, 7.41] | 3.62 [1.85, 7.08] |
| Breast | P=0.00024 | P=0.0073 | P=0.0001 | P=0.0002 |
| N=286 | K=24 |  | K=68 |  |
| NKI | 4.19 [1.83, 9.57] | 4.16 [1.78, 9.72] | 4.34 [1.89, 9.95] | 3.21 [1.32, 7.86] |
| Breast | P=0.0007 | P=0.001 | P=0.0005 | P=0.01 |
| N=295 | K=57 |  | K=13 |  |
| GSE3494 | 4.84 [1.72, 13.62] | 2.40 [0.85, 6.81] | 6.68 [1.97, 22.65] | 5.31 [1.57, 17.91] |
| Breast | P=0.0028 | P=0.1 | P=0.0023 | P=0.0072 |
| N=315 | K=5 |  | K=65 |  |
| Chung | 16.82 [1.97, 144.0] | 8.89 [0.58, 136.2 ] | 12.45 [1.31, 118.7] | 3.19 [0.44, 23.33] |
| HN | P=0.01 | P=0.12 | P=0.028 | P=0.25 |
| N=60 | K=2 |  | K=14 |  |
| Beer | 45.99 [5.87, 360.2] | 5.63 [1.06, 29.81] | 8.67 [1.30, 57.65] | 5.01 [0.64, 39.23] |
| Lung | P=0.0003 | P=0.042 | P=0.025 | P=0.13 |
| N=86 | K=11 |  | K=9 |  |
| GSE4573 | 4.34 [1.78, 10.59] | 1.88 [0.78, 4.51] | 3.77 [1.66, 8.57] | 1.89 [0.85, 4.20] |
| Lung | P=0.0013 | P=0.16 | P=0.0015 | P=0.12 |
| N=130 | K=10 |  | K=15 |  |

* For each dataset, all available clinical variables were included in the models; a reduced model of significant clinical variables was derived using backward likelihood stepwise selection in a multivariate Cox model in each dataset; the variables in the reduced model are shown in Table 3 for each dataset. The hypoxia score of the signature was then introduced in this model.

^£^ Minimal prognostic signature as defined in Materials and Methods; hazard ratio (HR), low and higher 95% confidence intervals (CI), P value in the multivariate reduced Cox model and number of genes, K, are shown.
